# Development of an ANCA-Associated Vasculitides Patient Registry in Greece

**DOI:** 10.31138/mjr.31.1.84

**Published:** 2020-03-31

**Authors:** Konstantinos Thomas, Alexandros Panagiotopoulos, Aggelos Banos, Evangelia Argyriou, Kyriaki Boki, Dimitrios Boumpas, Dimitrios Vassilopoulos

**Affiliations:** 1 Joint Rheumatology Program, National and Kapodistrian University of Athens, School of Medicine, Clinical Immunology-Rheumatology Unit,; 2 Department of Medicine and Laboratory, Athens, Greece, Joint Rheumatology Program, National and Kapodistrian University of Athens, School of Medicine-Clinical Immunology-Rheumatology Unit, 4^th^ Department of Medicine, Athens, Greece,; 3 Rheumatology Unit, Sismanogleio General Hospital, Athens, Greece

**Keywords:** vasculitis, registry, comorbidities

## Abstract

Despite the recent advances in treatment, antineutrophil cytoplasmic antibody (ANCA) associated vasculitides (AAV) are among the rheumatic diseases with the highest morbidity and mortality. These outcomes are affected by a variety of factors apart from the disease itself, and are driven by infections, cardiovascular disease and drug toxicity. Even after the induction of remission, patients and their treating physicians are challenged by frequent relapses, accrual of chronic damage and impaired quality of life. Given their rarity along with their heterogeneous disease spectrum, patient registries are of utmost need for the study of all aspects of AAV. Already established AAV registries have a significant contribution in the apprehension of these diseases outside the strict context of clinical trials, and are becoming increasingly important as new drugs and treatment strategies are about to be introduced in daily practice. We here describe the design of a contemporary, web-based and easy-to-use patient registry specifically for patients with AAV, including all the necessary domains suggested by international working groups. This project is anticipated to contribute in better understanding of AAV in our country, with a future prospect of contributing to and sharing data with other international registries.

## INTRODUCTION

Antineutrophil cytoplasmic antibody (ANCA) associated vasculitides (AAV) include granulomatosis with polyangiitis (GPA), microscopic polyangiitis (MPA) and eosinophilic granulomatosis with polyangiitis (EGPA) and carry the highest mortality and morbidity among systemic vasculitides. Even when aggressively treated with glucocorticoids (GCs) and traditional immunosuppressives, mortality is still high (~25% at five years).^1,2^ Only a minority of deaths is directly attributed to vasculitis, while 50% of them are treatment-related and mainly caused by infections.^1^ We recently reported that in a cohort of 56 AAV Greek patients, the incidence of serious infections was 7.2 per 100 patient-years, reaching 20 per 100 patient-years in those with severe combined lung-kidney involvement.^3^ Biologic therapies have been an unprecedented breakthrough in the field of rheumatology therapeutics, and AAV could not be an exception. B-cell depletion (rituximab) is considered a first line therapy for induction and maintenance of remission in patients with GPA and MPA today.^4,5^ However, new issues have emerged, given the similar rates of serious infections with cyclophosphamide-based regimens,^4^ optimal (regular versus biomarker-tailored) dosing, and the management of patients who relapse while on treatment. Significant research projects on AAV are ongoing or recently completed and published, investigating the role of the novel oral, selective C5a receptor inhibitor avacopan as a steroid-sparing agent^6^ or clarifying the exact role of plasma exchange and lower dose corticosteroids during induction of remission.^7^

Nevertheless, the impact and uptake of these promising strategies will need to be studied in real-life settings outside the strict boundaries of clinical trials. Although current therapeutic regimens induce remission in the great majority of patients (70–90%), disease relapses are quite common (~40% in 5 years),^2^ especially in patients with lung involvement, positive PR3 autoantibodies, and high damage index, while it has been shown that the number of relapses correlates with the development of chronic lesions. Prevention, early diagnosis and management of vasculitis-associated damage have been recognised as of paramount importance. Damage in ANCA vasculitis occurs early in the course of the disease, varies according to the type of vasculitis, is correlated with both the disease itself and the treatment the disease itself and the treatment, and significantly affect prognosis.^8^

There are limited data in the literature regarding the long-term prognosis, comorbidities and mortality of patients with AAV in daily clinical practice, outside randomised trials and their extensions. Moreover, the complexity of these diseases in terms of differential diagnosis, the need for interdisciplinary collaboration, the treatment and assessment of outcomes and the long-term vigilance for relapses makes the management of AAV patients quite cumbersome. Due to the rarity of the disease, establishment of patient registries are crucial for the better understanding of the natural course of AAV and, interestingly, registries are one of fields of interest of the EUVAS Research Council along with disease assessment, biomarker studies, epidemiology and aetiology, clinical trials, genetics, toxicity and infection, database, and histology.

The OMERACT (Outcome Measures in Rheumatology) Vasculitis Working Group has recently proposed a core set of outcome measures in AAV research in order to align data collection and assure homogeneity of different datasets. This set includes at least assessment of disease activity, damage, patient-reported outcomes and mortality.^9^ Furthermore, the working group proceeded in the development of AAV-specific patient-reported outcome (PRO) after taking into account and incorporating patients’ perceptive of the impact of the disease on quality of life (AAV-PRO)^10^ and the implementation of these tools in registries of real-life patients is imperative. Recently, patient registries across Europe and the United States have provided useful information in that direction. Most of these registries are web-based, and this design enables easy access and data entry of participating centers.^11^

## AIM OF THE STUDY

To design and establish a Greek Registry, where patients with AAV (GPA, MPA, EGPA) will be registered and prospectively followed. Candidate participating centres will be rheumatology, nephrology and pulmonary clinics or departments with an expertise in the clinical care of patients with AAV.

## METHODS

The registry will include patients with GPA, MPA and EGPA that fulfil 2012 Chapel Hill Consensus Conference Disease Classification Criteria. Patients will be registered irrespective of their age, in order for rare cases of paediatric patients with ANCA vasculitis to be included. Informed consent will be obtained from all study subjects. Patients will be retrospectively and prospectively registered and evaluated at baseline, then every 6 months or at relapse (confirmed or suspected), and data will be entered into an electronic web-based platform. For any new registration, the user will be asked to enter epidemiologic data, a clinical assessment of disease activity (BVASv.3, BVAS/WG) and severity/chronicity (VDI), patient-reported outcomes (AAVPRO), comorbidities, hospitalisations (except from those caused by infections), infections (irrespective of severity), laboratory measurements, biopsy and imaging reports and medications. Especially for hospitalisations, infections and medications, universal coding is used to assure optimal data entry. In addition, biologic samples storage (Biobank) is designed, including information on storage of peripheral blood, serum, PBMCs, mRNA in order to enable future biomarker- or basic research projects.

## ANTICIPATED BENEFITS

AAV are a diverse group of diseases that, although rare, carry high mortality and morbidity and several unmet needs. This study is anticipated to reflect the current clinical and laboratory spectrum of patients with AAV in Greece, to describe treatment regimens currently used and compliance of treating physicians with guidelines, to document rates of chronic lesions and complications related to disease itself or immunosuppressive therapy, to assess efficacy, tolerance and complications of novel biologic agents, and create a biobank of biologic samples from different phases of the disease. Finally, a significant prospect of this study includes the future incorporation of this registry with similar international ones and its participation in multicentre studies held by the vasculitis community.
